# Use of Long-Term Care Decreased Over Time Among the Oldest-Old With and Without Dementia - A Register-Based Study

**DOI:** 10.1093/geroni/igab046.3547

**Published:** 2021-12-17

**Authors:** Mari Aaltonen, Leena Forma, Jutta Pulkki, Jani Raitanen, Marja Jylhä

**Affiliations:** 1 Tampere University, Tampere University / Tampere, Pirkanmaa, Finland; 2 University of Helsinki, University of Helsinki / Helsinki, Uusimaa, Finland

## Abstract

Care policies for older adults emphasize aging-in-place and home care over residential long-term care (LTC). We explore how the use of residential LTC in the last five years of life among people with and without dementia changed between those who died in 2001, 2007, 2013, and 2017 in Finland. Retrospective data drawn from the national health and social care registers include all those who died aged 70+ in 2007, 2013, and 2017, plus a 40% random sample from 2001 (N=128 050). Negative binomial regression analysis was used to estimate the association of dementia with LTC use during the last five years of life (1825 days). The independent variables included dementia, age, marital status, annual income, education, and chronic conditions. In the total study population, the proportion of LTC users and the mean number of days in LTC increased until 2013, after which it decreased. Changes in LTC use differed between different age groups and by dementia status. Over time, the decrease in round-the-clock LTC use was steep in those aged 90≤ with dementia and in people aged 80≤ without dementia. The individual factors related to morbidity and sociodemographic factors did not explain these results. The changes in LTC care policy may have contributed to the decrease in LTC use among the oldest. However, according to national statistics, the availability of formal home care has not increased. This development may suggest that the oldest-old and those with dementia – a highly vulnerable group – are left without proper care.

